# The Epigenetic Dimension of Protein Structure Is an Intrinsic Weakness of the AlphaFold Program

**DOI:** 10.3390/biom12101527

**Published:** 2022-10-20

**Authors:** Fodil Azzaz, Nouara Yahi, Henri Chahinian, Jacques Fantini

**Affiliations:** Department of Biology, INSERM UMR_S 1072, Aix-Marseille Université, 13015 Marseille, France

**Keywords:** alphafold, AI, protein structure, lipid rafts, ganglioside, membrane, therapy, molecular modeling, pathology

## Abstract

One of the most important lessons we have learned from sequencing the human genome is that not all proteins have a 3D structure. In fact, a large part of the human proteome is made up of intrinsically disordered proteins (IDPs) which can adopt multiple structures, and therefore, multiple functions, depending on the ligands with which they interact. Under these conditions, one can wonder about the value of algorithms developed for predicting the structure of proteins, in particular AlphaFold, an AI which claims to have solved the problem of protein structure. In a recent study, we highlighted a particular weakness of AlphaFold for membrane proteins. Based on this observation, we have proposed a paradigm, referred to as “Epigenetic Dimension of Protein Structure” (EDPS), which takes into account all environmental parameters that control the structure of a protein beyond the amino acid sequence (hence “epigenetic”). In this new study, we compare the reliability of the AlphaFold and Robetta algorithms’ predictions for a new set of membrane proteins involved in human pathologies. We found that Robetta was generally more accurate than AlphaFold for ascribing a membrane-compatible topology. Raft lipids (e.g., gangliosides), which control the structural dynamics of membrane protein structure through chaperone effects, were identified as major actors of the EDPS paradigm. We conclude that the epigenetic dimension of a protein structure is an intrinsic weakness of AI-based protein structure prediction, especially AlphaFold, which warrants further development.

## 1. Introduction

The elucidation of protein structures has been considered, for decades, to be the Grail of biology [1]. The major role played by proteins in biological mechanisms, and the assumption that the function of a protein is dependent on its three-dimensional structure, explains why this quest is one of biology’s major issues. The central dogma of molecular biology [2] includes a conceptual background, according to which, the structure of a protein is fully determined by its amino acid sequence, which is itself encoded in the genome [3]. Taking a dizzying shortcut, we can consider that the DNA sequence coding for a protein contains all the information required for its 3D structure, and thus, for its biological function [4]. In other words, protein structure is fully determined by its amino acid sequence [5]. If this assumption is correct, then with sufficient calculation capabilities, an artificial intelligence would be able to predict the 3D structure of a protein from sequence data. This is precisely what AlphaFold, a machine learning method, has recently announced [6]. Indeed, AlphaFold has made available the entire human proteome, and the 3D structure of any human protein can be downloaded freely from the European Bioinformatics Institute database (https://www.ebi.ac.uk, accessed on 1 September 2022) and from the Uniprot server (https://www.uniprot.org, accessed on 1 September 2022).

But can we really say that “the problem of protein structure has been solved”, as was announced by many media, social networks, and even top scientific journals (https://www.science.org/content/article/game-has-changed-ai-triumphs-solving-protein-structures, accessed 1 September 2022) following the publication of AlphaFold’s results on the human proteome ?

The success of AlphaFold is generally illustrated by the superposed images of a selected protein structure determined experimentally and predicted by AlphaFold [6] (https://www.deepmind.com/blog/alphafold-a-solution-to-a-50-year-old-grand-challenge-in-biology, accessed 1 September 2022). Although these images are very impressive, only an independent analysis of the protein structures made accessible by the algorithm can determine the value of the algorithm. Among the proteins with the most difficult 3D structures to elucidate, we can mention that the proteins localized in biological membranes are notoriously difficult to solve with x-ray crystallography [3]. However, these proteins are central to many human diseases; therefore, an accurate prediction of their structure is of high interest for drug design strategies. As AlphaFold is also claimed to excel at solving the 3D structure of membrane proteins [6], it is important to assess the reliability of the algorithm for this category of proteins.

We have already accomplished such analytical work with a first set of proteins comprising several membrane proteins [3]. This first study allowed us to observe that AlphaFold did not always give reliable results, revealing several inconsistencies in the location of the transmembrane domains controlling the typical topology of these membrane proteins. Additionally, we also observed that AlphaFold had difficulty in correctly predicting the structure of certain intra- and extracellular domains of several membrane proteins.

In this new study, we continue our analysis with a new sample of membrane proteins. We compare the results obtained by AlphaFold [6] with those of Robetta [7,8], another 3D structure prediction algorithm. We refine our study by showing that in most of the cases studied, the structures predicted by AlphaFold cannot be used as starting conditions for molecular docking analyses. These new results are discussed in light of the concept we developed in our previous publication, the “epigenetic dimension of protein structure” (EDPS) [3], which explains why AlphaFold fails to correctly predict the structure of membrane proteins. In contrast, we show that Robetta (which also provides an improved deep learning based modeling method, RoseTTAFold) [8] is generally superior to AlphaFold2 for the prediction of the structure of this particular class of proteins, and that Robetta structures are directly usable for molecular docking.

## 2. Methods

### 2.1. Membrane Proteins Study

The models of the membrane proteins predicted by AlphaFold2 for the epidermal growth factor receptor (EGFR), human synaptic vesicle glycoprotein C (h-SV2C), human synaptotagmin 1 (h-SYT1) and amyloid-beta precursor model (APP) were retrieved from uniprot (https://www.uniprot.org/, accessed on 1 September 2022) using the uniref codes **#P00533** for EGFR, **#Q496J9** for h-SV2C, **#P21579** for h-SYT1 and **#P05067** for APP. The ab-initio models were generated using the web-based service Robetta (https://robetta.bakerlab.org/, accessed on 1 September 2022). To make sure that the membrane proteins predicted by Robetta can be inserted into a planar lipid bilayer, each membrane protein was loaded on CHARMM-GUI and analyzed with the “bilayer builder” tool [9,10].

### 2.2. Structural and Functional Study of the Luminal Domain of h-SV2C

To compare the structural reliability of the luminal domain of h-SV2C predicted by Robetta and AlphaFold2, we performed a structural alignment of each model with the luminal domain of h-SV2C resolved by Xray diffraction (PDB: 4JRA). Then, we merged the coordinates of botulinum neurotoxin A1 (BoNT/A1) with each model to obtain a complex BoNT/A1-h-SV2C for each h-SV2C structure predicted by Robetta and AlphaFold2. The complexes were submitted to energy minimization with the Polak-Ribière algorithm of HyperChem (CHARMM force field, 0.1 kcal/mol as the gradient conditions) in order to optimize the inter and intra-molecular contacts of the proteins. The energy of interaction of the minimized complexes were measured on Molegro Software using the tool “ligand energy inspector” (http://molexus.io/molegro-molecular-viewer/, accessed on 1 September 2022).

### 2.3. Soluble Proteins Study

AlphaFold2 predicted models for botulinum neurotoxin A (BoNT/A), botulinum neurotoxin B (BoNT/B) and complex factor H (CFH) were obtained from uniprot sequence data using the uniref **#P0DPI0** for BoNT/A, **#P10844** for BoNT/B and **#P08603** for CFH. To compare the inter-molecular contacts of each neurotoxin model in initial interaction with their membrane receptors, we docked them with their protein receptor, and we inserted the receptor into a lipid bilayer that mimics a lipid-raft environment.

### 2.4. Docking of BoNT/B with its Membrane Receptors

The synaptotagmin binding pocket surface of the crystal structure of BoNT/B (PDB: 2NP0) was manually docked to the surface of h-SYT1 in an initial orientation compatible with the membrane topology of h-SYT1 and surrounding ganglioside cofactors [11]. These initial conditions take into acccount the experimental data that identify the BoNT/B domains that bind to h-SYT1 and to the ganglioside cofactor [12,13,14]. Molecular details of these initial conditions are given in Figure S1.

### 2.5. TM-Score and Root-Mean-Square Deviation

The measurements of TM-score and Root-mean-square deviation (RMSD) for the comparison of the global structures were performed using the online tool “TM-score” available on Zhong Lab website (https://zhanggroup.org/TM-score/, accessed on 5 October 2022) by taking the experimental structure of the corresponding model as reference. Similar calculations were performed for the domain comparison by using the domain structure of AlphaFold2 and Robetta models as reference. Additionally, we computed the values of RMSD for the comparison of each global structure and each protein domain structure using PyMOL software (https://pymol.org/2/, accessed on 5 October 2022).

## 3. Results

### 3.1. EGFR

**Figure 1:**
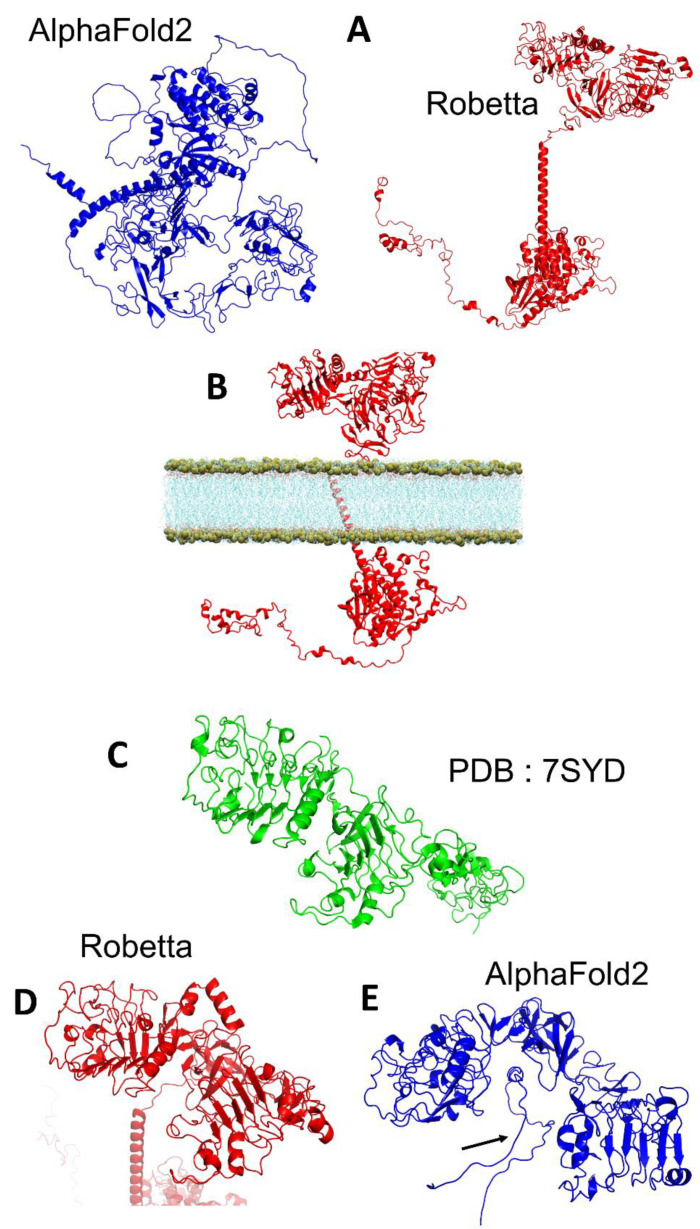
Comparison of the structure of the epidermal growth factor receptor retrieved from Alpha Fold (depicted as cartoon colored in blue) or modelized via Ab-initio calculation on Robetta (red) **(A)**. Molecular model of the insertion of the receptor obtained by Robetta in a lipid membrane environment (**B**). Comparison of the structure of the epidermal growth factor receptor resolved by Cryo-EM (PDB: 7SYD, resolved from the residue 25 to 638) ((**C**), green) with the structure obtained by Robetta ((**D**), red) and AlphaFold2 ((**E**), blue). In (**E**), the arrow points to an unstructured region that was predicted and inserted by the AlphaFold2 algorithm between the two extra-cellular domains.

In this first section, we compared the structure of four different membrane proteins predicted by AlphaFold2 and Robetta. Our first example is the epidermal growth factor receptor (EGFR), a signalling membrane receptor involved in several types of cancers [15]. The models generated by these algorithms are significantly different. Robetta could predict the correct spatial organization of the extra-cellular, transmembrane, and intra-cellular regions of EGFR whereas the structure predicted by AlphaFold2 is clearly not membrane compatible, but rather looks like a soluble protein (Figure 1A). One of the major challenges of predicted models is to know if they could be used for further molecular modeling work. To assess the reliability of EGFR, we attempted to insert this receptor into a membrane. The Figure 1B shows that the topology of EGFR predicted by Robetta is sufficiently accurate to insert this membrane protein in a lipid bilayer. The extracellular part of EGFR was previously solved by Cryo-EM from residues 25 to 638 (Figure 1C). The comparison of the crystal structure with Robetta and AlphaFold2 models revealed that the experimental structure matched better the prediction of Robetta (Figure 1D) than AlphaFold2 (Figure 1E). Additionally, we can see that AlphaFold2 has predicted that the unstructured 1151–1185 region of EGFR, which belongs to the intracellular domain of the receptor, is positioned extracellularly (Figure 1E, arrow). This could explain why AlphaFold2 failed to predict an accurate structure of EGFR that matches the spatial organization revealed by the experimental approach.

### 3.2. h-SV2C

**Figure 2:**
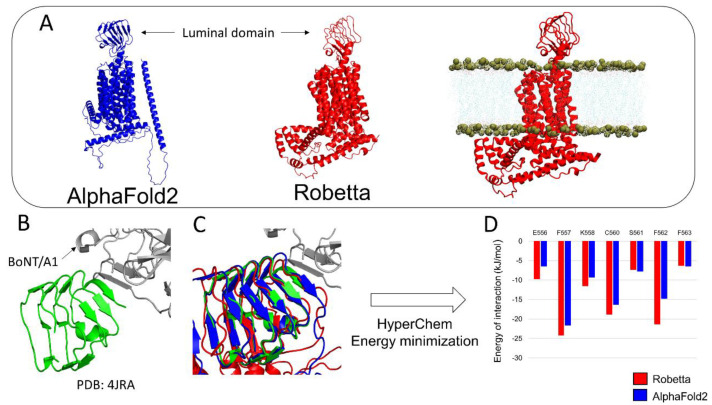
Comparison of the structure of human synaptic vesicle glycoprotein C (h-SV2C) predicted by AlphaFold2 (blue) and Robetta (red). Insertion of the model predicted by Robetta into a lipid bilayer (**A**). Crystal structure of the luminal domain of h-SV2C in complex with BoNT/A1 (**B**). Structural alignment of h-SV2C predicted by AlphaFold2 and Robetta with the crystal structure of h-SV2C (**C**). Comparison of the energy of interactions of each h-SV2C-BoNT/A1 complex (**D**).

Next, we challenged AlphaFold2 and Robetta to predict the structure of synaptic vesicle protein h-SV2C, a complex membrane protein with 12 transmembrane domains which plays a role in dopamine neurotransmission and Parkinson’s disease [16], and is also used as a receptor by botulinum neurotoxins [17]. The models are shown in Figure 2. Both AlphaFold2 and Robetta successfully modelized the luminal domain of h-SV2C and the 12 transmembrane regions. However, only Robetta was able to correctly predict the intracellular domain of h-SV2C. In contrast, AlphaFold2 predicted it to be a long alpha helix that crosses the membrane, which then makes it impossible to insert in a lipid bilayer as the Robetta model does (Figure 2A). Studies using the luminal domain of h-SV2C are of high interest because this structure serves as a membrane receptor for the botulinum neurotoxin BoNT/A1 [17], which is the most potent microbial neurotoxin in humans, with a lethal dose of 1 ng/kg [18,19]. The luminal domain of h-SV2C in complex with BoNT/A1 was previously resolved by Xray diffraction (Figure 2B). Topologically, both the AlphaFold2 and Robetta models proposed a structure similar to the experimental structure (Figure 2C). To study which of the AlphaFold2 or Robetta models is the most accurate for interacting with BoNT/A1, we docked the neurotoxin near the interaction site of each h-SV2C model and we submitted them to energy minimization. The energy of interaction of the major amino acid residues interacting with the BoNT/A1 are presented in Figure 2D.

**Figure 3:**
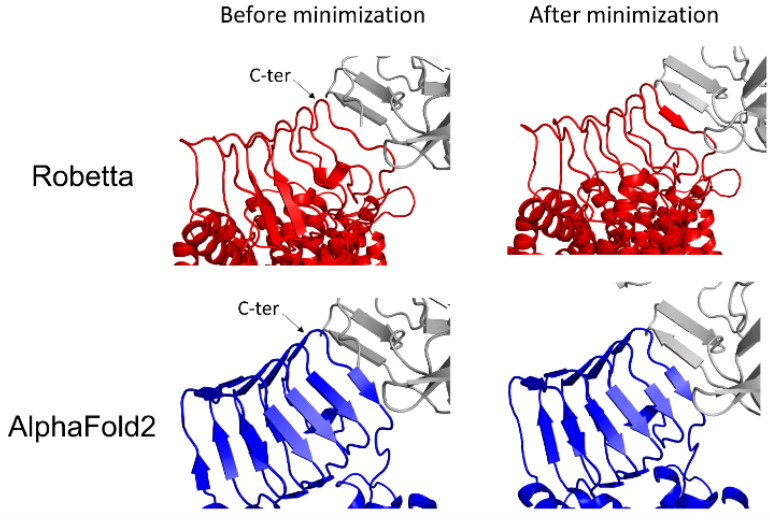
Structural conformational changes of BoNT/A1-h-SV2C complex involving the Robetta model (red) or the AlphaFold2 model (blue) after energy minimization.

Snapshots of the complexes before and after energy minimization are presented in Figure 3. The analysis of the energy of interaction obtained for each minimized complex suggests that the Robetta model is generally more accurate than the AlphaFold2 model because the Robetta model displayed a slightly higher energy of interaction for residue K558, and, to a lesser extent, for the residues E556, F557, C560 and F562 (Figure 2D). These differences are in good agreement with the structural details of each complex. Indeed, the C-terminal extremity of the luminal domain of h-SV2C of the Robetta model adapts its structure to interact with the beta strand structure of BoNT/A1, a conformational rearrangement that is not observed in the AlphaFold2 model (Figure 3).

### 3.3. h-SYT1 and APP

**Figure 4:**
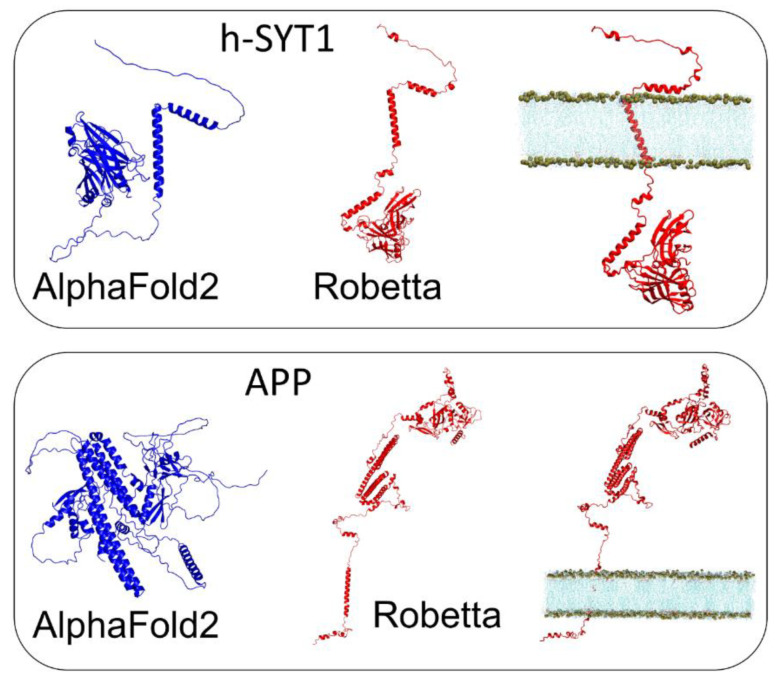
Comparison of the structure of human synaptotagmin 1 (h-SYT1) (**top panel**) and APP (**bottom panel**) retrieved from AlphaFold2 (blue) or generated by ab-initio modeling with Robetta (red).

Our third and fourth examples are human synaptotagmin-1 (h-SYT1), which is also known to interact with BoNT/A1 [13] and the Alzheimer’s amyloid precursor protein (APP), which, upon proteolytic cleavage, produces the Alzheimer’s β-amyloid peptide [20]. Both proteins have a single transmembrane domain. As in the case of EGFR, the AlphaFold2 models for h-SYT1 and APP looked more like globular proteins than membrane proteins (Figure 4). In contrast, Robetta predicted a spatial organization that was compatible with a membrane protein insertion in a lipid bilayer, and the clearcut presence of a luminal domain and an intracellular domain.

Taken together, these data suggested that Robetta is more accurate than AlphaFold2 for the prediction of membrane protein structure and topology.

### 3.4. BoNT/A1 and BoNT/B1

Since the AlphaFold2 algorithm suffers from obvious deficiencies concerning the relative spatial organization of the domains of a protein, we were interested in investigating the case of large soluble proteins that have several domains. For this purpose, we selected the microbial neurotoxins BoNT/A1 and BoNT/B1, and factor H of human complement (a regulatory cofactor for the protease factor I in the breakdown of C3b in the complement system of immune defence) [21]. For each selected protein, we compared the models obtained by AlphaFold2 and Robetta with the experimental structure solved by X-ray diffraction.

**Figure 5:**
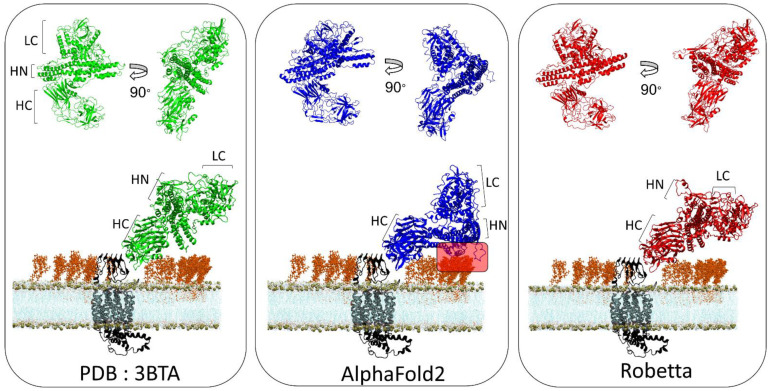
Structural comparison of the spatial organization of the domains of botulinum neurotoxin A obtained from Xray diffraction (PDB: 3BTA) (**left**), AlphaFold2 (**middle**) and Robetta (**right**) and molecular modeling of each structure with its membrane receptor human synaptic vesicle glycoprotein C (h-SV2C) in a neural membrane context. The toxin receptor h-SV2C is depicted as a cartoon colored in black. The phosphate atom of each POPC lipid is shown as brown spheres. GT1b molecules are represented as orange sticks and the lipid tail of POPC molecules are shown as thin blue lines.

Botulinum neurotoxins are composed of three domains, a light chain (LC) which has a metalloprotease activity, a translocation domain (HN) and a C-terminus heavy chain (HC) [22]. HC is the most studied domain because it is responsible for the recognition of toxin receptors on the extracellular surface of neural membranes. In the case of BoNT/A1, the spatial organization of the pattern predicted by the AlphaFold2 is different from that proposed by X-ray diffraction, while the Robetta model suggests a structure that is quite the same as the experimental one (Figure 5). It is known that BoNT/A1 uses, as membrane receptor, a complex between gangliosides (which form lipid raft domains in the plasma membrane) [23] and the luminal domain of synaptic vesicle glycoproteins h-SV2C [17,24,25]. To evaluate if these different structural organizations can induce a bias in the initial binding of BoNT/A1 with its membrane receptor, we docked each structure to the luminal domain of h-SV2C embedded in a lipid raft. As indicated by a red frame in Figure 5, a large surface of the HN domain of BoNT/A1 interacts with the sugar moiety of gangliosides, while this is not the case for the Robetta model and the experimental structure.

**Figure 6:**
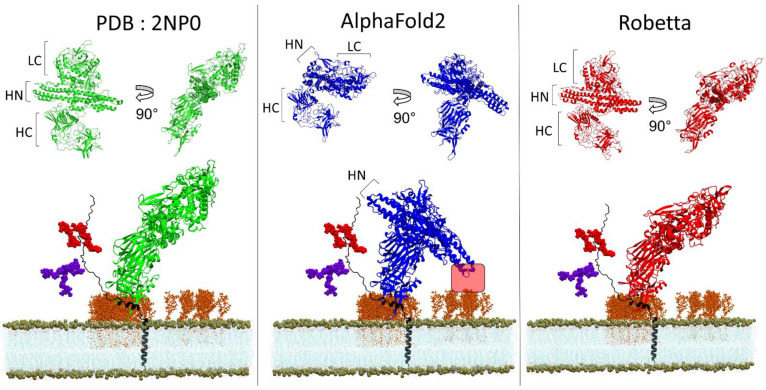
Structural comparison of the spatial organization of the domains of botulinum neurotoxin B obtained from Xray diffraction (PDB: 2NP0) (**left**), AlphaFold2 (**middle**) and Robetta (**right**) and molecular modeling of each structure with its membrane receptor human synaptotagmin 1 in a neural membrane context. The toxin receptor h-SYT1 is depicted as cartoon colored in black. The phosphate atom of each POPC lipids is shown as brown spheres. The GT1b molecules are represented as orange sticks, the lipid tails of POPC molecules are shown as thin blue lines and the N-glycan and O-glycan of h-SYT1 are depicted as purple and red spheres respectively.

Next, we were interested in performing a similar evaluation for BoNT/B1, since this serotype is the second most potent in humans, just after BoNT/A1. To this end, we docked BoNT/B1 to its protein receptor h-SYT1 [26] and we inserted the complex into a lipid bilayer that mimics a lipid raft context. As for BoNT/A1, the structural organization of the HN and LC domains in the AlphaFold2 model are different from the experimental structure, while the Robetta model displays a similar spatial organization (Figure 6). As a result, the extremity of the HN domain of the AlphaFold2 model is wrongly positioned within the 5Å distance (red frame in Figure 6), which could allow an interaction with the sugar moiety of gangliosides, compared to the Robetta model and the experimentally determined structure, for which this option is not possible.

**Figure 7:**
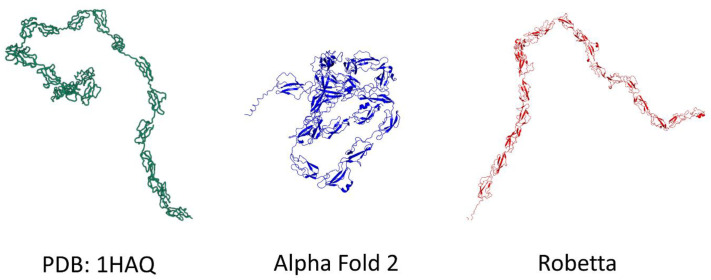
Comparison of Xray diffraction (PDB: 1HAQ), AlphaFold2 and Robetta models for complement factor H. In the case of this highly flexible protein, AlphaFold2 predicts a globular shape whereas Robetta’s model is rather elongated.

Finally, we compared the models of the complement factor H, a protein which adopts a typical serpentine shape [21], as illustrated by the experimental model solved by solution scattering (PDB: 1HAQ) (Figure 7). The Robetta algorithm managed to propose an elongated serpentine folding for this protein, while AlphaFold2 proposed a condensed wool ball structure similar to those proposed for EGFR and APP (Figure 7). These data indicated that the AlphaFold2 algorithm has difficulties in predicting elongated structures, which is an important feature for the prediction of a correct spatial organization of proteins, such as botulinum neurotoxin and membrane proteins, as we show across our examples. Interestingly, the failure of AlphaFold2 in this case was not due to a low confidence in its prediction, since the pLDDT [6] of this model was in the 70–90 range (Figure S2). In any case, this deficiency significantly impacts the prospect of using the AlphaFold2 model to perform molecular modeling of such flexible proteins.

### 3.5. TM-Score and Root-Mean-Square Deviation of AlphaFold2 and Robetta Models

The reliability of Robetta and AlphaFold2 predictions can be estimated, respectively, by the Å error estimate and pLDDT values. The data for the set of proteins analyzed in the present study are available in Figures S2–S4. As expected, the level of confidence of both programs was higher for structured vs. unstructured regions. Overall, all the proteins had high pLDDT values, so that the diffences between the predictions of AlphaFold and Robetta are not due to a bias in the selection of the models. Moreover, to further compare the predictions of AlphaFold2 and Robetta with the available experimental structures, we performed a systematic analysis of the TM-score and RMSD values (Table 1).

We specifically assessed the spatial organization of each protein by comparing the structures predicted by AlphaFold2 and Robetta, using the extracellular region of EGFR (Figure S5), the intracellular region of h-SYT1 (Figure S6), full length BoNT/A (Figure S7), full-length BoNT/B (Figure S8) and APP (Figure S9) as corresponding references. In this analysis, each individual domain predicted by AlphaFold2 or Robetta was compared with the corresponding experimental structure.

The data in Table 1 indicated that Robetta models match the spatial organization of experimental structure domains better than AlphaFold2 models, as demonstrated by a lower RMSD and a higher TM-score. However, when each domain is taken individually, AlphaFold2 models present a lower RMSD and a higher TM-score value than Robetta models, suggesting that AlphaFold2 is more accurate for predicting the folding of protein domains.

### 3.6. A Chaperone Activity in Lipid Rafts

**Figure 8:**
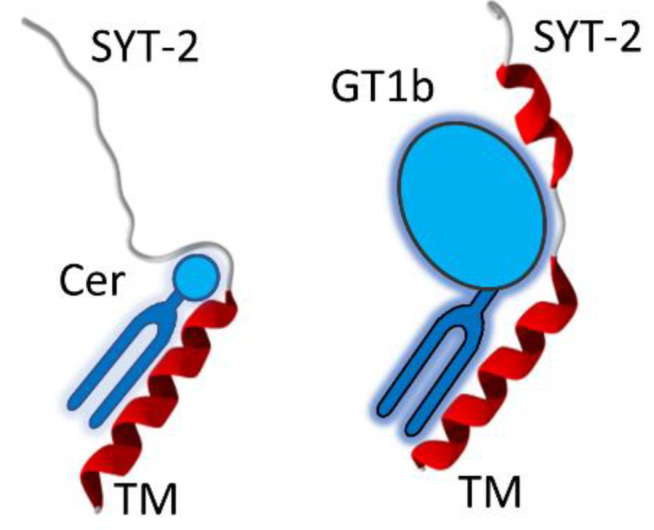
Lipid chaperone effect as a major parameter of the EDPS paradigm. The extracellular domain of synaptotagmin-2 (h-SYT2) is totally disordered when bound to ceramide (Cer), whereas it acquires a α-helix structure when bound to ganglioside GT1b. Both models were obtained with Hyperchem and submitted to energy minimization with the Polak–Ribière algorithm according to the protocol used for BoNT/A1-h-SV2C as described in Materials and Methods. Lipid molecular structures have been schematized for clarity. The global shape and volume of these lipids are directly responsible for these typical chaperone effects. This mechanism accounts for the critical role played by raft lipids on protein structure, illustrating the EDPS paradigm. TM, transmembrane domain.

Lipid rafts contain different sphingolipid species, which have been shown to control the conformation of proteins, so that they are considered to be lipid chaperones [27]. A typical example of this chaperone activity is presented in Figure 8. It concerns synaptotagmins, which, like SV2 glycoproteins, can be used as membrane receptors by botulinum neurotoxins [13]. Synaptotagmins are associated with lipid rafts in synaptic vesicles and on the plasma membrane of neural cells [28,29,30]. When the protein interacts with small raft lipids, such as the regulatory signal transduction molecule ceramide, its extracellular domain remains disordered. In this case, the polar head group of ceramide has a small area of interaction with the protein, and thus, has a limited effect on its shape. In contrast, the ganglioside GT1b has a large saccharidic polar head group that establishes numerous contacts with the protein and drives the α-helix folding of the first part of its extracellular domain. Thus, despite the fact that this part of synaptotagmin is predicted to adopt a helical shape by both Robetta and AlphaFold2 (Figure 4), this α-helix structure can collapse under the reversible control of chaperone raft lipids, which have the power to transform a functional protein (α-helix conformer) into a nonfunctional one (disordered conformer). In fact, only the synaptotagmin-GT1b complex behaves as a functional receptor for botulinum toxin [11], which obviously cannot be predicted on the sole basis of the amino acid sequence of synaptotagmin.

## 4. Discussion

The prediction of the 3D structure of proteins based on their amino acid sequence has made considerable progress in recent years with the advent of methods based on deep learning [1]. However, despite these advances, we should take into account that many problems still need to be solved [3]. First of all, the working hypothesis according to which all proteins necessarily have a three-dimensional structure has been contradicted by the results of the sequencing of the human genome [31]. In fact, there is a very large part of the human proteome comprising intrinsically disordered proteins (IDPs), i.e., proteins having no ordered structure, or having at least one disordered part [32]. Thanks to genome sequencing data and prediction algorithms, it has been estimated that IDPs represent about 40% of all proteins in eukaryotes, constituting the “unfoldome”, which corresponds to the set of disordered proteins [31]. IDPs are involved in the regulation of key biological functions including signal transduction, gene expression, cell division, differentiation and inflammation [33]. An intriguing aspect of IDPs is their capacity to adapt their conformation to their environment [34]. In this case, the Anfisen rule “one amino acid sequence, one structure, one function” becomes “one amino acid sequence, numerous structures, numerous functions”. Among the disease-associated proteins that contain chameleon [35] or discordant sequences [36], and thus, that can adopt distinct structures [37], Aβ, α-synuclein and tau are of critical importance, since their conformational plasticity is directly related to the pathological mechanisms of neurological disorders [38]. Not surprisingly, the case of these proteins has been identified as serious limitation of AlphaFold [3,37].

The case of synaptotagmin (Figure 8) is also emblematic, since the extracellular part of this membrane protein can remain disordered or be partially structured as an α-helix, depending on the nature of the membrane lipid with which it interacts [11]. The consequence of this structuring is critical for the binding of botulinum neurotoxin which uses synaptotagmin as a membrane receptor. It is therefore very clear that in this case, the amino acid sequence alone does not provide all the information necessary to be able to predict a 3D structure. It is precisely this limitation of prediction algorithms that led us to develop the concept of “epigenetic dimension of protein structure” (EDPS) [3]. This paradigm takes into account the influence of the protein environment, which, in addition to the amino acid sequence, imposes folding constraints. However, it excludes common post-translational modifications of proteins, such as phosphorylation, glycosylation or lipidation, that can be predicted from consensus amino acid motifs [3]. Membrane proteins are a perfect example of this paradigm, as specific lipids act as key cofactors (i.e., chaperones) for protein folding and stability [27,38,39,40,41,42]. The existence of protein-lipid “co-structures” have been identified as an issue for the heterologous expression of membrane proteins [43]. Indeed, injecting the information of an amino acid sequence into a heterologous cell does not warrant correct expression and folding of a membrane protein if the specific lipid requirements of this particular protein are not respected. Yet, in some instances, water can play a similar role as membrane lipids on protein folding. In the aqueous extracellular space, the initially disordered amyloid protein A β_1-42_, folds into a typical β-structure, consistent with the propensity of its amino acid sequence, to adopt a secondary β-strand organization [36]. Then, in a lipid environment, the same protein may be forced to adopt a helical structure [44,45,46,47], which is thus non-“natural”, but rather induced by the environment, consistent with the EDPS paradigm. A similar mechanism applies for the cellular prion protein, a typical α/β discordant protein [20,36] which is stabilized by raft lipids in a physiological α-helical structure [20,38], whereas it can switch to the pathological β-structure when those protective lipids detach from the protein [38]. Clearly, these proteins pose a serious problem to AI-based prediction methods of protein structure based on the amino acid sequence.

The intrinsic environmental limitations highlighted by the EDPS paradigm [3] are illustrated in the present study by numerous examples of membrane proteins for which AlphaFold does not provide correct structures. On the other hand, we find that Robetta’s results take much better account of membrane topology, at least for the set of proteins analyzed. At this point, we have not clearly identified what gives Robetta this advantage over AlphaFold, the latter stumbling on the difficulty of predicting a realistic membrane topology, but also having a problem for loop structures [48]. Both algorithms are based on the functioning of neural networks. In a recent comparative study focused on G-protein-coupled receptors (GPCR), Lee et al. [49] concluded that the popular template-based method Modeler is superior to both AlphaFold and Robetta (RosseTTAfold) when good templates are available. The best AlphaFold models matched closely to crystal structures, but Robetta was generally more accurate. In any case, we hope that our study will allow the developers of AlphaFold to take into account new parameters for membrane proteins and improve the algorithm accordingly. The stakes are very high because many human pathologies involve membrane proteins, including receptors and ion channels. The design of molecules binding to these proteins using in silico docking approaches can only be done if reliable 3D structures are available. If we consider the case of the APP protein, which is the precursor of the β-amyloid peptide of Alzheimer’s disease, we cannot use the 3D structure proposed by AlphaFold2 because it absolutely does not respect membrane topology (Figure 4). Another difficulty appears if we now consider alpha-synuclein, the protein responsible for Parkinson’s disease. α-Synuclein is an IDP [50] that can be secreted by nerve cells [51,52]. A part of secreted α-synuclein can be attracted by selected gangliosides in lipid rafts domains, which, with the assistance of cholesterol, triggers the structuration and oligomerization of Ca^2+^ permeable pores (amyloid pores) [45,46,53]. A similar molecular mechanism also applies for Alzheimer’s Aβ_1–42_, a typical IDP which also forms amyloid pores once inserted in the plasma membrane of brain cells [45,46,54]. For both α-synuclein and Aβ_1-42_, structure predictions are necessarily inaccurate, since these proteins can exist in a myriad of conformations and various oligomeric assemblies [37], until they reach the plasma membrane, where gangliosides and cholesterol have the opportunity to force them to adopt a precise structure, making the formation of a calibrated amyloid pore possible [20]. To circumvent this difficulty, which complicates the implementation of a therapeutic strategy, Fantini and Yahi have created a therapeutic peptide (AmyP53) targeting gangliosides, and thus, preventing any interaction of α-synuclein or Aβ at the membrane level [55]. The design of this peptide took into account the concept of IDPs by adapting it to a synthetic molecule. This was possible by applying the EDPS paradigm, and more precisely, by considering the chaperone role of membrane lipids on the structure of proteins, beyond the single amino acid sequence [56]. However, the whole process first required the elucidation of α-synuclein and Aβ_1-42_ binding to brain gangliosides at the molecular level [38,57,58].

Another major outcome of the present study concerns the reliability of structure protein prediction for molecular docking. We took the example of SV2, which, like synaptotagmin, is also used as membrane receptor by botulinum neurotoxins. The design of inhibitors of this toxin requires deciphering, at the molecular level, the mechanisms involved in the toxin-cell interaction. X-ray diffraction studies are incomplete, as they can only be performed with the extracellular part of the receptors. It is, therefore, crucial to obtain structural data of these proteins in their entirety and in their membrane environment. Our study has highlighted inconsistencies in Alpha-Fold predictions which do not provide a correct initial structure for docking the toxin on SV2.

Overall, although performed on a limited set of proteins, our study shows that the Achilles heel of 3D membrane protein structure prediction algorithms is indeed the mode of interaction of these proteins with the membrane bilayer. This intrinsic limitation requires reconsidering the global paradigm that links the amino acid sequence of a protein and its 3D structure. All proteins do not necessarily have a 3D structure, and the environment brings its own set of parameters that must be integrated into the algorithms. Indeed, it would be illusory to think that a single algorithm could predict the structure of all proteins. This would amount to considering that the folding of membrane proteins and their topology in biological membranes obeys the same rules as those which structure water-soluble proteins. On the opposite of this deterministic view of biology, the EDPS paradigm considers water and membrane lipids as key parameters that act “beyond the genetic code” (hence “epigenetic”) and give a degree of conformational freedom for distinctive proteins. Until now, the only parameter implicitly considered, and therefore not mentioned, was water. A fundamental mistake in biology has been to think for too long that this parameter applied to the entire proteome of a living organism. Indeed, protein secondary structure predictions have been established from the structure of water-soluble proteins and then extrapolated to any type of protein [59]. This confusion seems to be reproduced with AlphaFold, which, without diminishing its performance, cannot achieve the impossible, i.e., assign a structure to proteins which do not have one, or extrapolate the parameters controlling the structure of a water-soluble protein to a membrane protein.

We are of course aware that the parameters controlling the 3D structure of proteins, apart from the amino acid sequence, have been identified several years ago [3]. The data accumulated on amyloid proteins, alpha/beta transitions and IDPs are all exceptions to Anfinsen’s dogma according to which the amino acid sequence of a protein contains all the information necessary for its folding. Nevertheless, we want to draw the attention of non-experts to the fact that these data call into question the very principle on which AlphaFold is based, i.e., the predictions of 3D structure from sequence data. Under these conditions, grouping together all these exceptions to the dogma under the same term EDPS seems, to us, to be an important clarification of the scientific value of programs such as AlphaFold. The above-mentioned chaperone activity, gangliosides, is a perfect illustration of the need to introduce this paradigm. According to the scientific literature, this conformational effect can be either α-helix [60,61] or β-sheet [62,63] structuration, depending on the protein concerned. Additionally, even for the same protein, the type of secondary structure induced by gangliosides can vary dramatically from α to β, according to the protein-ganglioside ratio [62,64,65]. By grouping all these phenomena under the same term, EDPS, we clearly limit the field of application of AlphaFold to proteins having a stable 3D structure based on an architectural organization built on a predictable secondary structure, a field in which AlphaFold generally excels [6]. However, when it comes to applying the rules established for soluble proteins to membrane proteins [59], it is difficult for AlphaFold to achieve success. Indeed, proline and glycine, which are very rare in the helices of water-soluble proteins, are overrepresented in helical transmembrane domains, giving these domains possibilities of regulation that exist only in membrane proteins [66]. These are, therefore, indeed functional epigenetic phenomena, in the sense that these phenomena depend on the environment of the proteins, and not only on the amino acid sequence coded in the genes. The living retains a share of anarchy, which, for the moment, remains totally unpredictable.

## Figures and Tables

**Table 1:** Root mean square deviation (RMSD) and template-modeling score (TM-score) values for each AlphaFold2 and Robetta model according to their corresponding experimental structures. The RMSD is the measure of the average distance (in Å) between the backbone atoms of superimposed proteins. TM-score lies between 0 and 1, where 1 indicates a perfect match between two structures (thus the closer to 1 the better).

Protein	Template PDB	RMSD Robetta	RMSD AlphaFold2	TM-Score Robetta	Tm-Score AlphaFold2
**EGFR (25–638)**	7SYD	21.1	27.085	0.29	0.23
**EGFR (25–309)**	7SYD	2.64	2.36	0.86	0.91
**EGFR (366–492)**	7SYD	0.88	0.44	0.96	0.99
**h-SYT1 (141–419)**	2R83	12.19	17.482	0.44	0.41
**h-SYT1 (143–265)**	2R83	1.31	1.27	0.93	0.95
**h-SYT1 (274–419)**	2R83	0.88	0.47	0.95	0.97
**APP (30–123)**	1MWP	1.08	0.6	0.90	0.92
**APP (290–342)**	1APP	1.2	0.57	0.85	0.95
**BoNT/A (whole protein)**	3BTA	4.327	25.47	0.77	0.61
**BoNT/A LC (0–441)**	3BTA	2	0.7	0.92	0.97
**BoNT/A HN (442–850)**	3BTA	2.46	1.58	0.9	0.95
**BoNT/A HC (851–end)**	3BTA	2.02	1.55	0.92	0.96
**BoNT/B (whole protein)**	2NP0	5.298	22.558	0.78	0.684
**BoNT/B LC (0–441)**	2NP0	1.75	1.41	0.96	0.97
**BoNT/B HN (442–850)**	2NP0	2.56	1.98	0.91	0.95
**BoNT/B HC (851–end)**	2NP0	2.37	1.65	0.91	0.96

## Data Availability

Not applicable.

## Supplementary Materials

The following supporting information can be downloaded at: https://www.mdpi.com/article/10.3390/biom12101527/s1, Figure S1: Close view into the molecular mechanism of interaction of the HC domain of BoNT/B with its membrane receptor after molecular docking process; Figure S2: Angstrom error estimate for Robetta models and pLDDT score for AlphaFold2 models for soluble proteins BoNT/A, BoNT/B and CFH; Figure S3: Angstrom error estimate for Robetta models and pLDDT score for AlphaFold2 models for the membrane protein EGFR and h-SV2C; Figure S4: Angstrom error estimate for Robetta models and pLDDT score for AlphaFold2 models for h-SYT1 and APP; Figure S5: RMSD and TM-score values of AlphaFold2 and Robetta models for EGFR taking as template the Cryo-EM structure in PDB file 7SYD; Figure S6: TM-score and RMSD values of AlphaFold2 and Robetta models for h-SYT1 taking as template the coordinates of the Xray structure stored in PDB file 2R83; Figure S7: TM-score and RMSD values of AlphaFold2 and Robetta models for BoNT/A taking as template the coordinates of the Xray structure in PDB: 3BTA; Figure S8: TM-score and RMSD values of AlphaFold2 and Robetta models for BoNT/B taking as template the coordinates of the Xray structure in PDB: 2NP0; Figure S9: TM-score and RMSD values of AlphaFold2 and Robetta models for APP taking as template the coordinates of the Xray structure stored in PDB files 1MWP for residues 30 to 123 or 1APP for residues 290 to 342.
