# Supplementary material for: The Epigenetic Dimension of Protein Structure Is an Intrinsic Weakness of the AlphaFold Program
Source: Biomolecules. 2022 Oct 20;12(10):1527. doi: 10.3390/biom12101527 (PMC9599222; doi:10.3390/biom12101527)
Supplement: Supplementary file 1 [file biomolecules-12-01527-s001.zip › biomolecules-1957842-supplementary.pdf]

Supplementary information

The epigenetic dimension of protein structure is an intrinsic weakness of the AlphaFold program

Fodil Azzaz, Nouara Yahi, Henri Chahinian and Jacques Fantini
Aix-Marseille Université and INSERM UMR_S 1072, Marseille, France.

Corresponding author: Jacques Fantini, e-mail: jacques.fantini@univ-amu.fr

Content : Figures S1 to S9

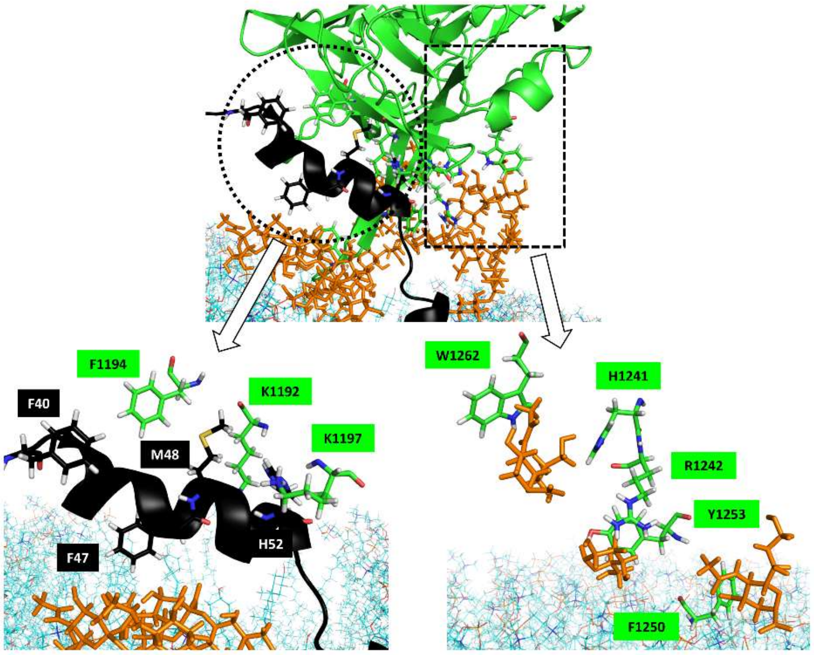

Figure S1

Figure S1 : Close view into the molecular mechanism of interaction of the HC domain of BoNT/B with its membrane receptor after molecular docking process. The initial binding of the toxin consists of interacting with h-SYT1 via its synaptotagmin binding pocket with the key amino acid residues K1192, F1197 and F1194 and in the same time interacting with the sugar moiety of gangliosides via its ganglioside binding site (key residues H1241 and W1262) and its lipid binding loop (key residues R1242, F1250 and Y1253).

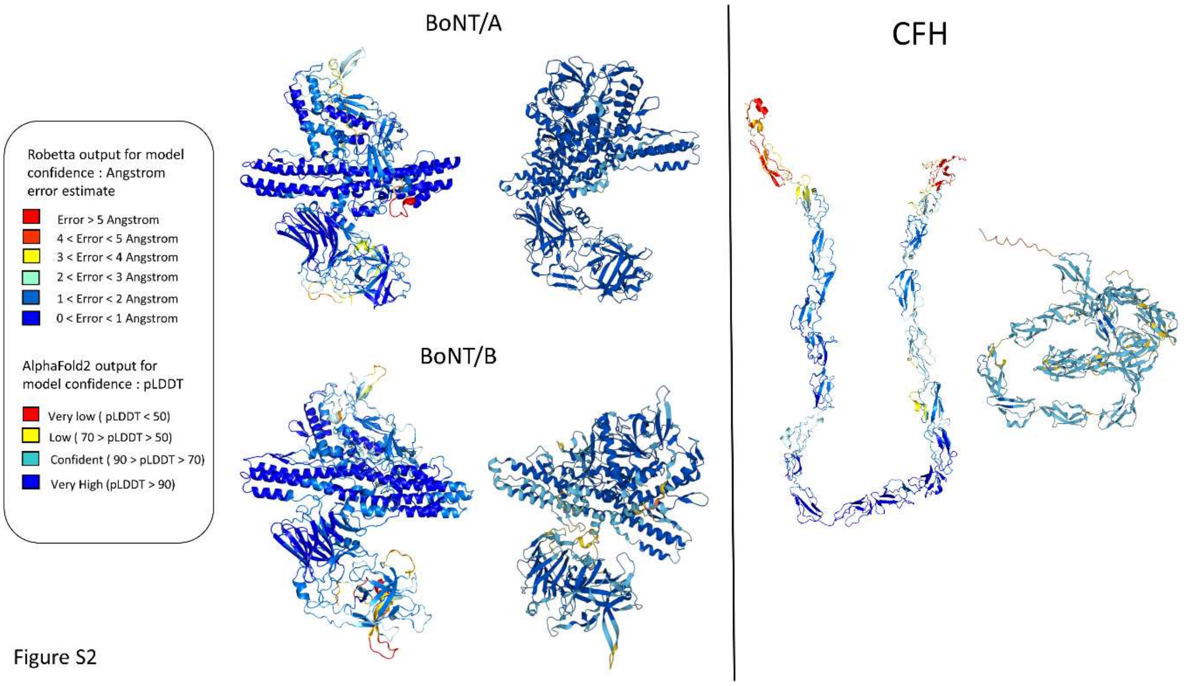

Figure S2 : Angstrom error estimate for Robetta models and pLDDT score for AlphaFold2 models for soluble proteins BoNT/A, BoNT/B and CFH. The legend of each color is given in the figure.

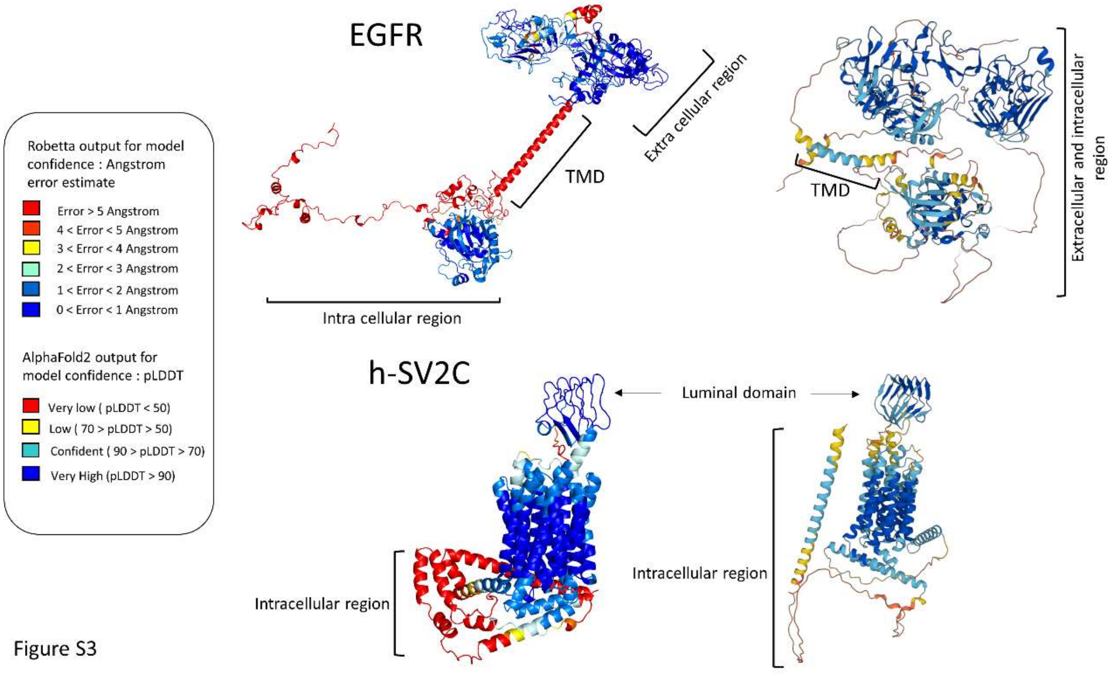

Figure S3 : Angstrom error estimate for Robetta models and pLDDT score for AlphaFold2 models for the membrane protein EGFR and h-SV2C. The range of values are shown as colored gradient on the structure.

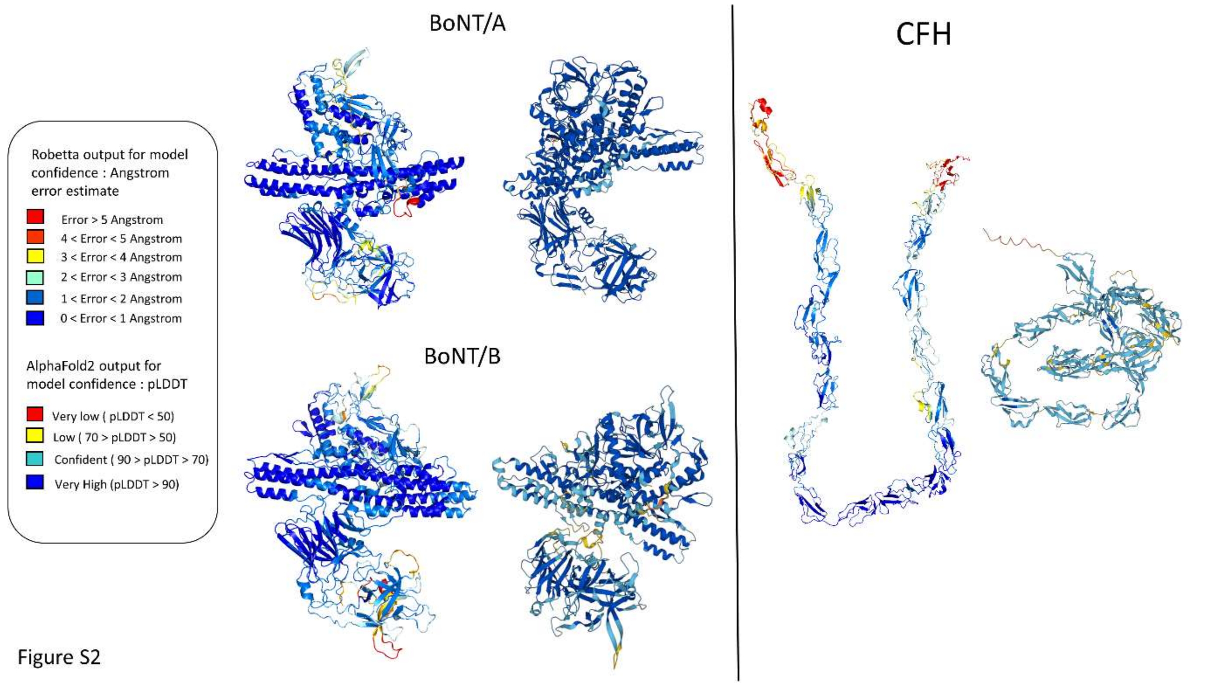

Figure S4 : Angstrom error estimate for Robetta models and pLDDT score for AlphaFold2 models for h-SYT1 and APP.

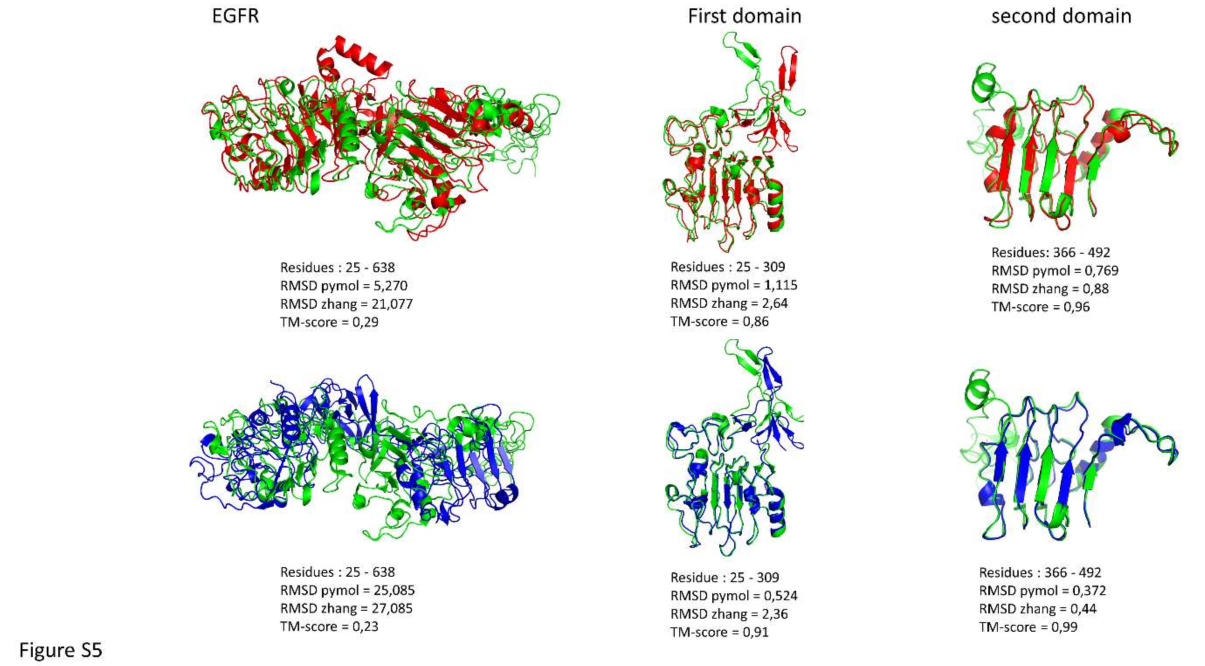

Figure S5 : RMSD and TM-score values of AlphaFold2 and Robetta models for EGFR taking as template the Cryo-EM structure in PDB file 7SYD.

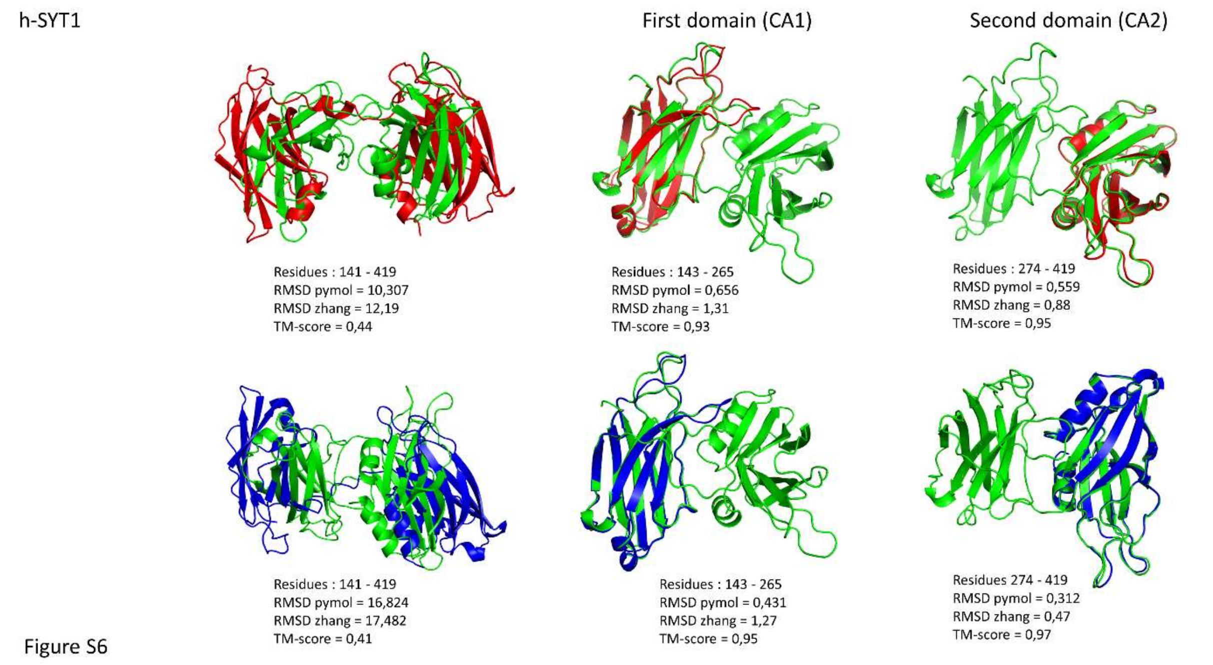

Figure S6 : TM-score and RMSD values of AlphaFold2 and Robetta models for h-SYT1 taking as template the coordinates of the Xray structure stored in PDB file 2R83. Each set of values is accompanied of a snapshot showing the structural alignment of the corresponding model with the experimental template. Same color code as Figure S1.

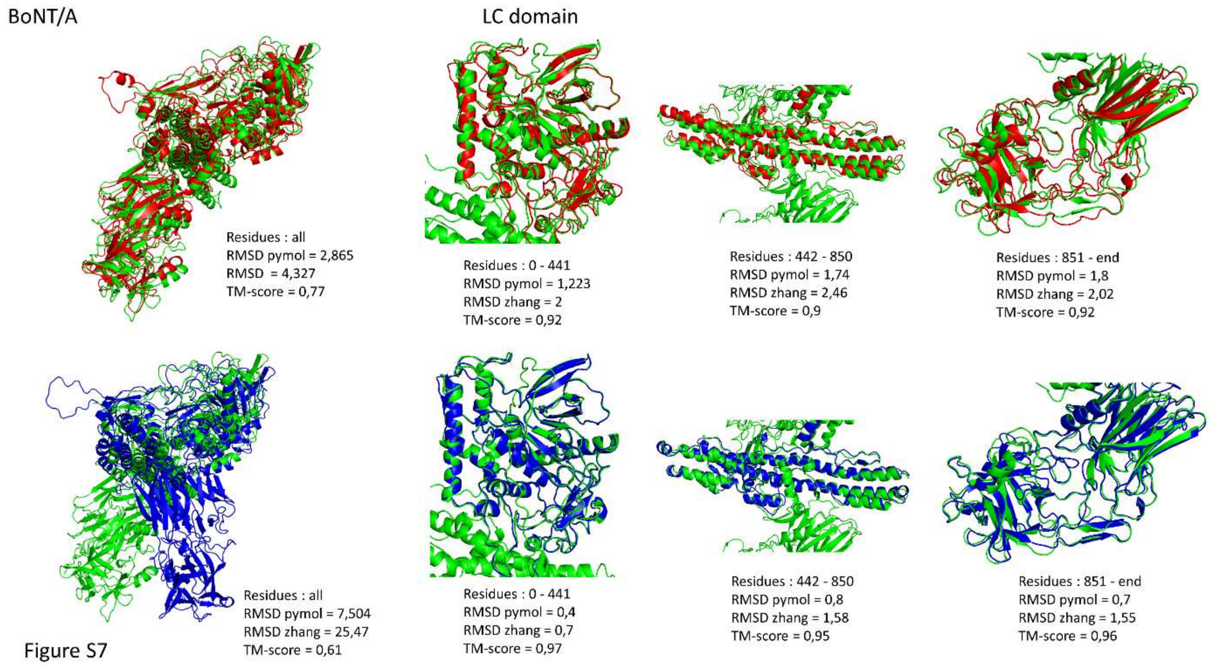

Figure S7 : TM-score and RMSD values of AlphaFold2 and Robetta models for BoNT/A taking as template the coordinates of the Xray structure in PDB : 3BTA. Each set of values is accompanied with a snapshot showing the structural alignment of the corresponding model with the experimental template. Same color code as Figure S1.

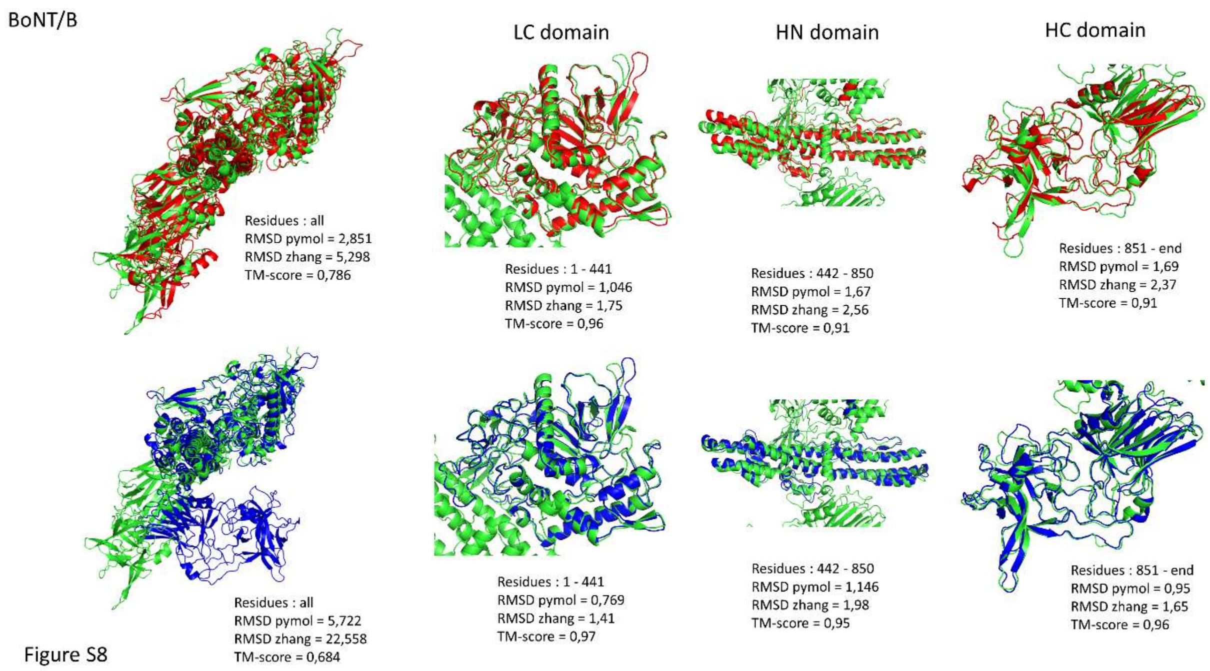

Figure S8 : TM-score and RMSD values of AlphaFold2 and Robetta models for BoNT/B taking as template the coordinates of the Xray structure in PDB : 2NP0. Each set of values is accompanied of a snapshot showing the structural alignment of the corresponding model with the experimental template. Same color code as Figure S1.

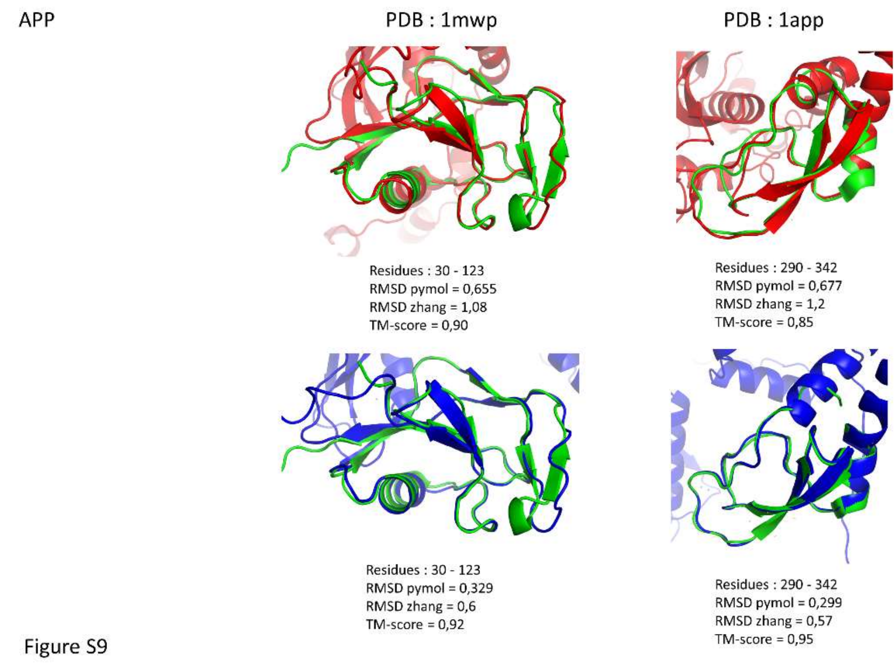

Figure S9 : TM-score and RMSD values of AlphaFold2 and Robetta models for APP taking as template the coordinates of the Xray structure stored in PDB files 1MWP for residues 30 to 123 or 1APP for residues 290 to 342. Each set of values is accompanied of a snapshot showing the structural alignment of the corresponding model with the experimental template. Same color code as Figure S1.
